# CSF1R Protein Expression in Reactive Lymphoid Tissues and Lymphoma: Its Relevance in Classical Hodgkin Lymphoma

**DOI:** 10.1371/journal.pone.0125203

**Published:** 2015-06-12

**Authors:** Ana M. Martín-Moreno, Giovanna Roncador, Lorena Maestre, Elena Mata, Scherezade Jiménez, Jorge L. Martínez-Torrecuadrada, Ana I. Reyes-García, Carmen Rubio, José F. Tomás, Mónica Estévez, Karen Pulford, Miguel A. Piris, Juan F. García

**Affiliations:** 1 Pathology Department, MD Anderson Cancer Center, Madrid, Spain; 2 Monoclonal Antibodies Unit, Spanish National Cancer Centre (CNIO), Madrid, Spain; 3 Proteomics Unit, Spanish National Cancer Centre (CNIO), Madrid, Spain; 4 Haematology Department, MD Anderson Cancer Center, Madrid, Spain; 5 Nuffield Division of Clinical Laboratory Sciences, Radcliffe Department of Medicine, University of Oxford, John Radcliffe Hospital, Oxford, United Kingdom; 6 Pathology Department, Hospital Universitario Marqués de Valdecilla, Santander, Spain; University of Nebraska Medical Center, UNITED STATES

## Abstract

Tumour-associated macrophages (TAMs) have been associated with survival in classic Hodgkin lymphoma (cHL) and other lymphoma types. The maturation and differentiation of tissue macrophages depends upon interactions between colony-stimulating factor 1 receptor (CSF1R) and its ligands. There remains, however, a lack of consistent information on CSF1R expression in TAMs. A new monoclonal antibody, FER216, was generated to investigate CSF1R protein distribution in formalin fixed tissue samples from 24 reactive lymphoid tissues and 187 different lymphoma types. We also analysed the distribution of CSF1R+, CD68+ and CD163+ macrophages by double immunostaining, and studied the relationship between CSF1R expression and survival in an independent series of 249 cHL patients. CSF1R+ TAMs were less frequent in B-cell lymphocytic leukaemia and lymphoblastic B-cell lymphoma than in diffuse large B-cell lymphoma, peripheral T-cell lymphoma, angioimmunoblastic T-cell lymphoma and cHL. HRS cells in cHL and, with the exception of three cases of anaplastic large cell lymphoma, the neoplastic cells in NHLs, lacked detectable CSF1R protein. A CSF1R+ enriched microenvironment in cHL was associated with shorter survival in an independent series of 249 cHL patients. CSF1R pathway activation was evident in the cHL and inactivation of this pathway could be a potential therapeutic target in cHL cases.

## Introduction

The presence of a characteristic inflammatory microenvironment is a fundamental component of the tumour mass in classical Hodgkin lymphoma (cHL) [1]. In most cases, the neoplastic Hodgkin-Reed-Sternberg (HRS) cells are in a minority, being greatly outnumbered by non-neoplastic cells such as lymphocytes, macrophages, eosinophils, mast cells, fibroblasts, microvessels and other stromal elements.

In recent years this microenvironment, largely recruited by the HRS cells through the secretion of a variety of chemokines and cytokines, has been shown to play an essential role in pathogenesis [2,3]. It can supply the tumour cells with growth factors, inhibit antitumour immune responses, and in turn be perpetuated by additional factors secreted by other reactive cells. Consistent with this, there have been several reports of the association between specific cell subpopulations of the microenvironment and clinical outcome. For example, increased cytotoxic T cells (Granzyme B+ / TIA1+)[4] and polarized Th2 cells (PD-1+) [5] have been associated with worse outcome and shorter survival. In contrast, an increased number of FOXP3+ regulatory T cells (Tregs) has been related to better outcome and longer survival [4,6].

Additionally, tumour-associated macrophages (TAMs) have been associated with clinical outcome and survival in cHL. Initial gene expression analyses indicated an association between macrophage signatures and treatment response[4,7,8,9]. Studies using both gene expression and immunohistochemical (IHC) techniques of macrophages have revealed a correlation between decreased TAM frequency and improved clinical outcome[10]. Although several further IHC articles have reported comparable results[11,12], others have found no association and highlighted problems arising from technical heterogeneity[13,14,15]. Variation in macrophage phenotypes, such as M1 and M2 [16] as well as other factors influencing the composition of the microenvironment, such as age and the presence of Epstein-Barr virus (EBV), may affect clinical presentation and outcome [8,12] and represent additional confounding factors.

The functional relevance of the microenvironment and the identification of prognostic biomarkers have also been described in non-Hodgkin lymphomas (NHLs), mainly in follicular lymphoma (FL) [6,17,18,19,20] and diffuse large B-cell lymphoma (DLBCL) [6]. The majority of these studies rely on IHC analyses that use specific T-cell or macrophage markers. Despite these advances, there remains a lack of detailed functional descriptions of the interactions between tumour cells and immune elements of the microenvironment.

The maturation and differentiation of tissue macrophages depends upon the activation of tightly regulated pathways. Macrophage colony stimulating factor or colony stimulating factor-1 (CSF1) binds to the high-affinity receptor tyrosine kinase, the cFMS/CSF1 receptor (CSF1R) [21] and is a key cytokine involved in the recruitment and activation of tissue macrophages [22]. Aberrant expression of *CSF1R* mRNA has been described in cHL-derived cell lines using reverse transcription (RT)-PCR and mRNA *in situ* hybridization [23,24]. Since the CSF1/CSF1R pathway could be inhibited by small molecules [25,26] and some *in vitro* studies also suggest that CSF1R could be an interesting therapeutic target for cHL [25], further analyses of the cell elements involved in the pathway are essential.

Despite increasing evidence of the role of TAMs in the lymphoma microenvironment, there is a lack of consistent information about the distribution of the CSF1R protein in normal and neoplastic tissues. Here we describe for the first time, the protein expression and cell distribution of CSF1R using a thoroughly validated new monoclonal antibody (mAb) in a large series of normal lymphoid tissues and major lymphoma types, with particular emphasis on cHL.

## Materials and Methods

### Production of anti-CSF1R monoclonal antibody

A new anti-CSF1R mAb (clone FER216) was produced by immunizing BALB/c mice with the extracellular domain of CSF1R fused to Fc fragment (ecCSF1R-Fc) prepared using a mammalian cell expression system. Complete details of the various procedures for protein production, purification, mouse immunization, hybridoma production and clone selection are available in S1 File.

An episomal expression system based on Epstein-Barr virus (EBV) components was used for recombinant CSF1R production in mammalian cells (HEK293). To this end, the sequence encoding the extracellular domain of CSF1R (NP_005202.2, residues 1–514) was fused to a C-terminal human IgG1 Fc sequence in a CMV-based pTT3 vector which features the EBV oriP replicator to allow episomal replication of the plasmid within cells. The protein was produced transiently in HEK293-EBNA cells, expressing the EBV nuclear antigen (EBNA). After 5 days post-transfection, secreted CSF1R-Fc was purified from the clarified growth media using a Protein A column (GE Healthcare) connected to an ÄKTA-prime system (GE Healthcare).

Two BALB/c mice were injected intraperitoneally (three times at 14-day intervals) with 100 μg of CSF1R-Fc fusion protein and Complete Freund's adjuvant (Difco).

The specificity of the mAb was confirmed by IHC and western blotting using either cytospin preparations or cell pellets of Fc-tagged human ecCSF1R expressed in HEK293 cells as described in S1 File.

All the experimental procedures involving the use of mice have been done in compliance with the current directives of the Spanish real decree RD53/2013 of February 1 of 2013 that contains the basic guidelines to be applied for the protection of animal used for scientific research and education. All the experimental procedures performed in the present study have been evaluated and approved by the Internal Committee or Animal Care and Use (IACUC) of the CNIO, the external Ethical Committee of the Instituto de Salud Carlos III and the Comunidad Autonoma of Madrid.

The CNIO Animal Facility technicians and the members of the research team have an extended experience in the use of animal models for research and are familiarized with a proper implementation of the experimental work to reduce, refine and replace (RRR) the animal use. Humane endpoint criteria will be applied for animal sacrifice.

### Lymphoma samples and cell lines

The CSF1R mAb was initially studied using 24 reactive lymphoid tissue (RLT) samples (lymph nodes, tonsils, bone marrow, thymus, and spleen) and 187 different lymphoma types: 10 cases of B-lymphoblastic lymphomas (B-LBL), 20 B-cell chronic lymphocytic leukaemia (B-CLL), 18 follicular lymphomas (FCL), 20 mantle-cell lymphomas (MCL), 20 diffuse large B-cell lymphomas (DLBCL), 19 Burkitt lymphomas (BL), 5 mucosa-associated lymphomas (MALT), 8 T-cell lymphoblastic lymphomas (T-LBL), 15 peripheral T-cell lymphomas (PTCL), 15 T-cell angioimmunoblastic lymphomas (AITL), 7 anaplastic large cell lymphomas (ALCL) and 35 cHL.

249 cHL cases were also studied in a tissue microarray (TMA) constructed with duplicate cores from selected areas of each case using a Tissue Arrayer Device (Beecher Instrument, Silver Spring, MD, USA). Clinical data and follow-up details were available for all cases (Table A in S1 File). All the normal and tumour samples were retrospectively collected from the Biobank of MDACC Madrid, in accordance with the technical and ethical procedures of the Spanish National Tumour Bank Network, including anonymization processes. Approval was obtained from the institutional review board *Comite Ético de Investigación Clínica* (Ethical Commite of Clinical Research) from Ramon y Cajal Hospital, Madrid, Spain (ref. 354/12) and written informed consent was obtained following the protocol of the Declaration of Helsinki. All the patients had been treated with standard adriamycin-based regimens, mostly ABVD.

Five cHL-derived cell lines (L428, L1236, KMH2, L540 and HDLM2) and the HEK293 cell line (human embryonic kidney) used for transfection were obtained from the German Collection of Microorganisms and Cell Cultures (DSMZ, Braunschweigh, Germany).

### Immunohistochemistry, double immunoenzymatic, double immunofluorescence and non-isotopic in situ hybridization

Formalin-fixed, paraffin-embedded (FFPE) tissues from normal and tumour samples were included in several TMA blocks. IHC analyses and *in situ* hybridization for the Epstein–Barr virus (EBV) were performed on TMAs using heat-induced epitope retrieval and standard procedures. Antibodies sources and dilution are described in Table B in S1 File. Protein expression was quantified using an automated scan, Chroma Vision Systems- ACIS III (DAKO, Glostrup, Denmark) as previously described [13]. For survival analyses, all IHC markers were categorized as high or low, using the median expression as the cut-off.

The Bond Polymer Refine detection system (Leica Biosystems, Solms, Germany) was used for single and double immunoenzymatic labelling of FFPE tissues, using several normal lymphoid tissues and 10 cases of cHL. Double labelling, using a double immunoperoxidase technique or the immunoperoxidase technique combined with immunofluorescence, was also performed to assess the relationship between CSF1R+, CD68+, CD163+ and CD30+ cells. Complete details of the protocol and antibodies are available in S1 File.

### Western blot and Immunoprecipitation and Q-PCR

All detailed methodology is described in S1 File. Briefly, western blot (WB) analyses of CSF1R protein were performed using total protein extracted from normal tissues, cHL tumours and cHL cell lines, lysed in a buffer containing 50 mM Tris (tris (hydroxymethyl) aminomethane)-HCl, pH 7.5, 150 mM NaCl, 1% Igepal (Sigma Chemical) and protease inhibitors (Roche, Mannheim, Germany). The total lysates of each cell line were denatured by heating in Laemmli sample buffer, resolved on a 10% sodium dodecyl sulfate-polyacrylamide gel (SDS-PAGE) and transferred onto nitrocellulose membranes for 2 h. Membranes were incubated overnight with blocking solution (5% milk in PBS) and immunoblotted for 1 h at room temperature with anti-CSF1R (FER216) (diluted 1:10), anti-CSF1R (61701) and α-tubulin monoclonal antibody (1:500) (CNIO), followed by incubation with horseradish peroxidase-conjugated secondary antibody (DAKO, Glostrup, Denmark).

For Q-PCR, RNA was extracted from 5 cHL tumor samples and tonsils (frozen tissues), and cHL cell lines, using an RNAeasy mini kit (Qiagen, Venlo, Limburg, Netherlands). Gene expression of CSF1R and CSF1 ligand were analyzed by using TagMan gene expression assays (CSF1L Hs00174164_m1; CSF1R Hs00911250_m1) and GUSB as endogenous control. All reactions were performed using a CFX96 thermal cycler (Bio-Rad, Hercules, CA, USA).

### In-gel digestion and LC-MS/MS analysis

All methodology is described in S1 File.

### Analyses of the phosphorylation status of CSF1R

To demonstrate that the 150 kDa band detected in the cHL cases corresponded to a phosphorylated CSF1R protein we treated two cells extract of cHL cases and two tonsils extracts with lambda protein phosphatase. Detailed methodology is described in S1 File.

### Statistical analyses

The overall survival (OS) was estimated by the Kaplan–Meier method and the curves were compared using the log-rank test. Multivariate Cox regression models were used to establish independence.

Relationships between clinical parameters and the IHC variables were examined using either the Spearman test (continuous variables) or the Pearson chi-square test (categorical variables).

Differences were considered to be statistically significant for values of p<0.05. All statistical analyses were performed using SPSS version 17.0.

## Results

### Specificity of the anti-CSF1R FER216 mAb

**Fig 1:**
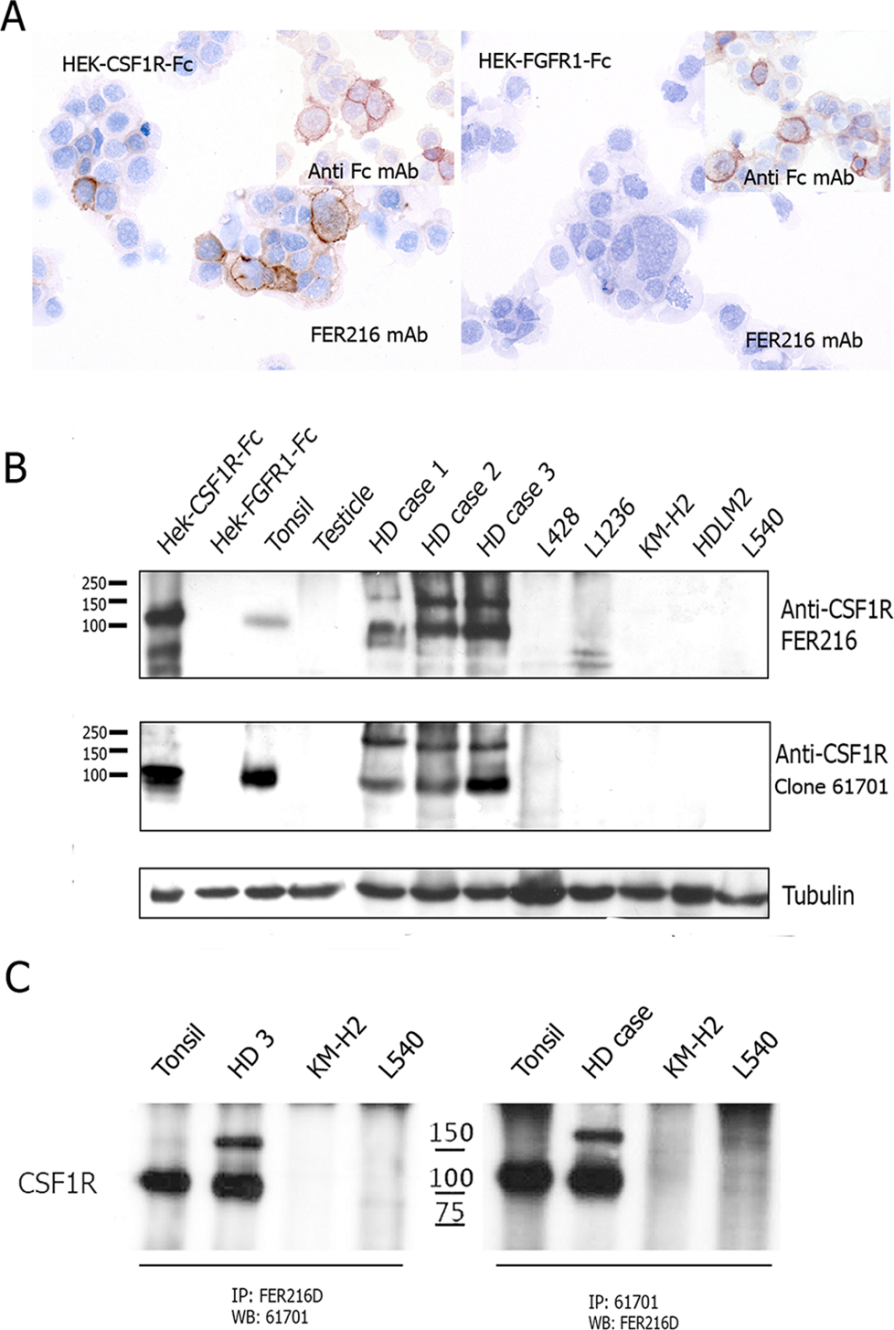
Immunohistochemistry, western blot and immunoprecipitation analyses of CSF1R. (A) IHC staining of antibody FER216 labelled the HEK-CSFR1-Fc transfectants expressing CSFR1 but not the negative control HEK-FGFR1-Fc expressing Fc transfectants. The positive anti-Fc mAb control labelled both sets of transfectants. (B) Western blot analyses of CSF1R protein using protein extracts from HEK293 cells transfected with either CSF1R or a closely related protein (FGFR1) as a negative control, normal tissues, cHL tumour samples, and cHL-derived cell lines. Antibody FER216 recognised a band of 100 kDa in tonsil extracts and two bands of 100 and 150 kDa in extract from three cHL biopsies. Comparable results were obtained using the anti-CSFR1 mAb clone 61701. The mAb to tubulin was used as the loading control. (C) IP followed by western blotting demonstrated comparable results using either the FER216 or Clone 61701. Both antibodies were able to immunoprecipitate bands of 100 kDa from tonsil and 100 and 150 kDa from a case of cHL.

IHC and WB studies showed the FER216 mAb recognised an Fc-tagged human ecCSF1R HEK293 transfectants (Fig 1A and 1B). In addition to the 100 kDa band in the transfectants, antibody FER216 also labelled a band of similar size in tonsil and three cHL cases. FER216 also strongly labelled a 150 kDa protein in the cHL cases (Fig 1B). Comparable results were obtained using the positive control antibody clone 61701(Fig 1B). Sequential IP and WB studies demonstrating that FER216 recognised proteins immunoprecipitated by clone 61701 further confirmed the specificity of FER216 for CSF1R (Fig 1C). The 150 kDa was absent or very faint in tonsil (Fig 1C). Both the 100 and 150 kDa protein bands were confirmed as corresponding to CSF1R protein by in-gel digestion and LC-MS/MS analysis, followed by peptide fragmentation and sequencing.

### Expression of CSF1R protein in normal lymphoid tissue and non-Hodgkin’s lymphomas

**Fig 2:**
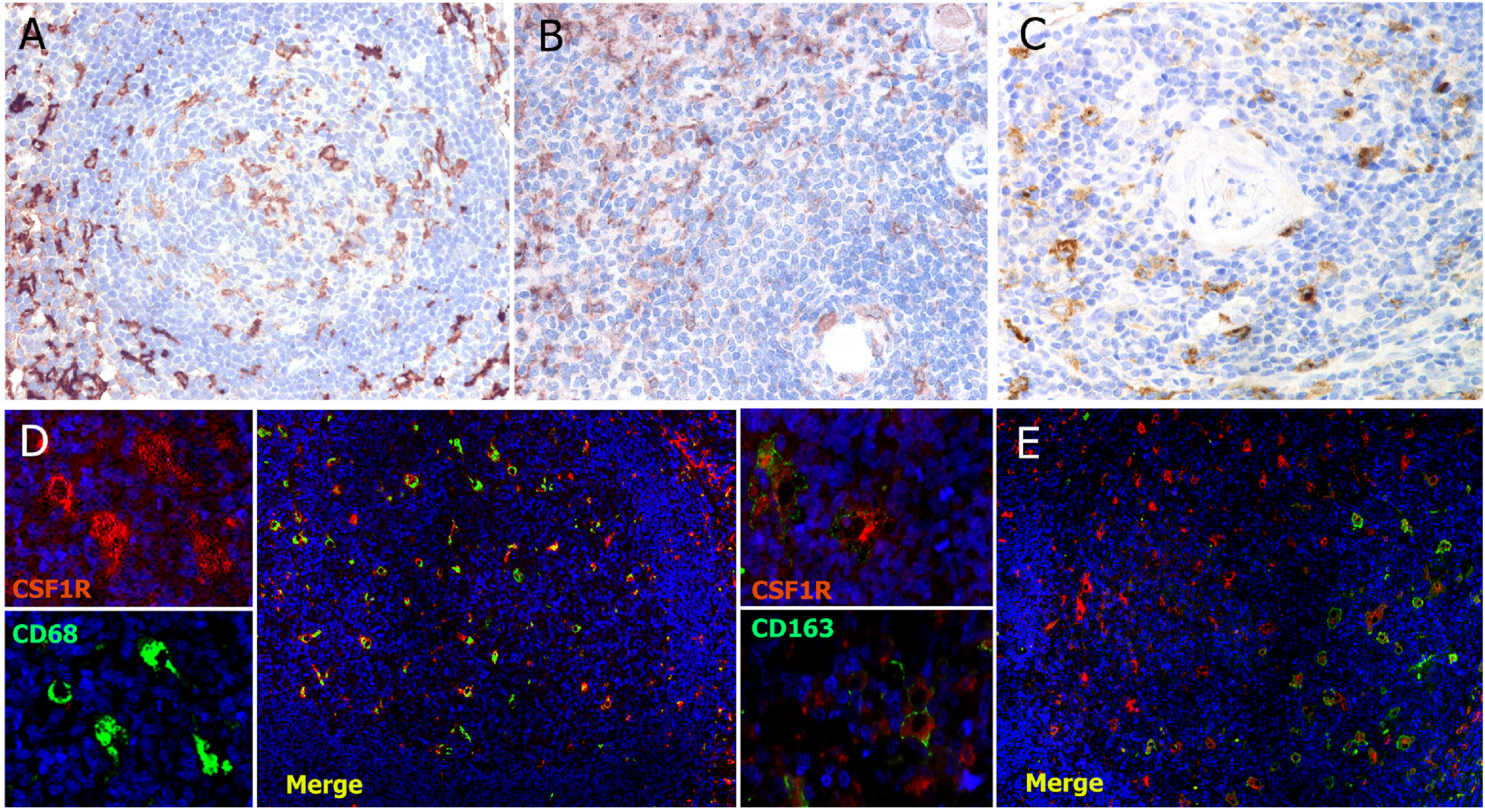
Immunohistochemical expression of CSF1R protein in normal lymphoid tissues. CSF1R+ macrophages (brown) are abundant in the paracortex of the lymph nodes (A), the red pulp of the spleen (B), and the medulla of the thymus (C). Panels D-E, double immunofluorescence experiments in tonsil: CSF1R (red) and CD68 (green) (D) and CSF1R (red) and CD163 (green) (E). It can be seen that while CSFR1+ cells also express CD68 (D), CD163 is limited to only a subpopulation of CSFR1+ cells (E).

The range of expression of CSF1R in RLT varied considerably between the different functional tissue compartments. The higher frequency of CSF1R+ macrophages was detected in the paracortex of lymph nodes and tonsils, the red pulp of the spleen and the medulla of the thymus (Fig 2, panels A-C). Double labelling studies confirm that while CSF1R was expressed in CD68+ macrophages (Fig 2D), only a subpopulation of the CSF1R+ cells also expressed CD163 (Fig 2D). This was more noticeable in the paracortical areas whereas germinal centre macrophages lacked CD163 (Fig 2E). This result is consistent with previous observations about monocyte differentiation, in which the *CSF1R* gene was not significantly differentially expressed between M1 versus M2 monocyte activation models [27].

The distribution of CSF1R protein in tonsil was also compared, using double immunoenzymatic labelling technique, with other markers of the microenvironment known to be associated with lymphoma pathogenesis, for example, FOXP3, PD-1 and the cytotoxic marker Granzyme B (Table C in S1 File). These analyses only revealed an association between CSF1R+ macrophages and FOXP3+ Tregs.

**Fig 3:**
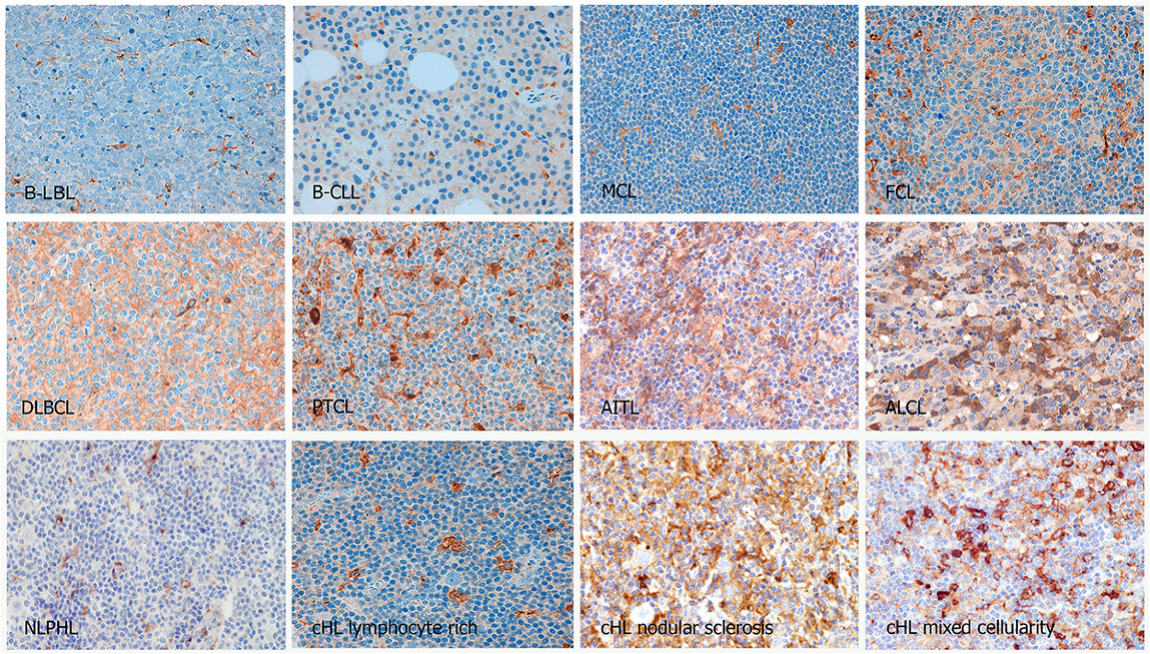
Immunohistochemical detection of CSF1R protein in different lymphoma types. CSF1R+ cells are less abundant in B-CLL and B- and T-LBL, and more frequent in the microenvironment of T-cell lymphomas (PTCL, AITL, and ALCL), and cHL.

The distribution of CSF1R+ cells varied greatly between the different lymphoma types and was restricted to macrophages and other monocytic cells in the microenvironment (Table 1; Fig 3). Automated quantification measurement showed fewer positive signals in B-CLL and B- and T-LBL, whereas more signals were identified in the microenvironment of T-cell lymphomas (PTCL, AITL, and ALCL), and cHL. Overall, this result was concordant with the results of manual cell counting (arbitrary cut-offs: fewer than 10%, 10–25%, or more than 25% positive cells; see Table 1). In general, most low-grade lymphomas had fewer CSF1R+ TAMs macrophages in their microenvironment, whereas DLBCL, T-cell lymphomas and cHL had higher CSF1R+ cell counts. Three of the 7 ALCL cases expressed CSF1R protein in some neoplastic cells.

**Table 1:** Distribution of CSF1R immunohistochemical expression in lymphomas.

Diagnosis			Low (<10%) *		Intermediate (10–25%) *		High (>25%) *	
Precursor Neoplasms	Nº cases	% Positive cells **	*Nº cases*	*%*	*Nº cases*	*%*	*Nº cases*	*%*
Precursor B lymphoblastic lymphoma	10	21.1	*4*	*40*,*0*	*3*	*30*,*0*	*3*	*30*,*0*
Precursor T lymphoblastic lymphoma	8	19.1	*2*	*25*,*0*	*3*	*37*,*5*	*3*	*37*,*5*
**Mature B Cell Neoplasms**								
Chronic lymphocytic leukemia	20	11.7	*10*	*50*,*0*	*7*	*35*,*0*	*3*	*15*,*0*
Follicular lymphoma	17	29.4	*10*	*58*,*8*	*2*	*11*,*8*	*5*	*29*,*4*
Mantle cell lymphoma	20	38.2	*7*	*35*,*0*	*3*	*15*,*0*	*10*	*50*,*0*
Diffuse large B cell lymphoma	20	50.9	*5*	*25*,*0*	*1*	*5*,*0*	*14*	*70*,*0*
Burkitt lymphoma	19	39.2	*6*	*31*,*6*	*5*	*26*,*3*	*8*	*42*,*1*
MALT lymphoma	5	23.3	*2*	*40*,*0*	*1*	*20*,*0*	*2*	*40*,*0*
**Mature T Cell Neoplasms**								
Peripheral T cell lymphoma	15	59.2	*1*	*6*,*7*	*2*	*13*,*3*	*12*	*80*,*0*
Angioimmunoblastic T cell lymphoma	14	51.5	*1*	*7*,*1*	*5*	*35*,*7*	*8*	*57*,*1*
Anaplastic large cell lymphoma***	7	42.6	*0*	*0*,*0*	*3*	*42*,*9*	*4*	*57*,*1*
**Hodgkin Lymphomas**								
Nodular lymphocyte-predominant HL	2	3.4	2	*100*,*0*	0	*0*,*0*	0	*0*,*0*
Lymphocyte-rich classic cHL	9	30.4	*2*	*22*,*2*	*5*	*55*,*6*	*2*	*22*,*2*
Nodular sclerosis cHL	131	47.5	*37*	*28*,*2*	*22*	*16*,*8*	*70*	*53*,*4*
Mixed cellularity cHL	49	39.0	*16*	*32*,*7*	*7*	*14*,*3*	*25*	*51*,*0*
**Total Hodgkin Lymphoma**	191	39.1	*57*	*29*,*8*	*34*	*17*,*8*	*97*	*50*,*8*

* Manual cell counting.

** Mean of results obtained from automated scanning of protein expression.

*** CSF1R is expressed in the tumour cells in 3/7 cases.

### CSF1R expression in cHL

In cHL, CSF1R+ TAMs were most frequent in the nodular sclerosis and mixed cellularity subtypes, with lower frequencies observed in lymphocyte-rich cHL (Table 1, Fig 3). Interestingly, CSF1R+ cells were almost absent from the microenvironment of the two cases of nodular lymphocyte-predominant HL studied.

**Fig 4:**
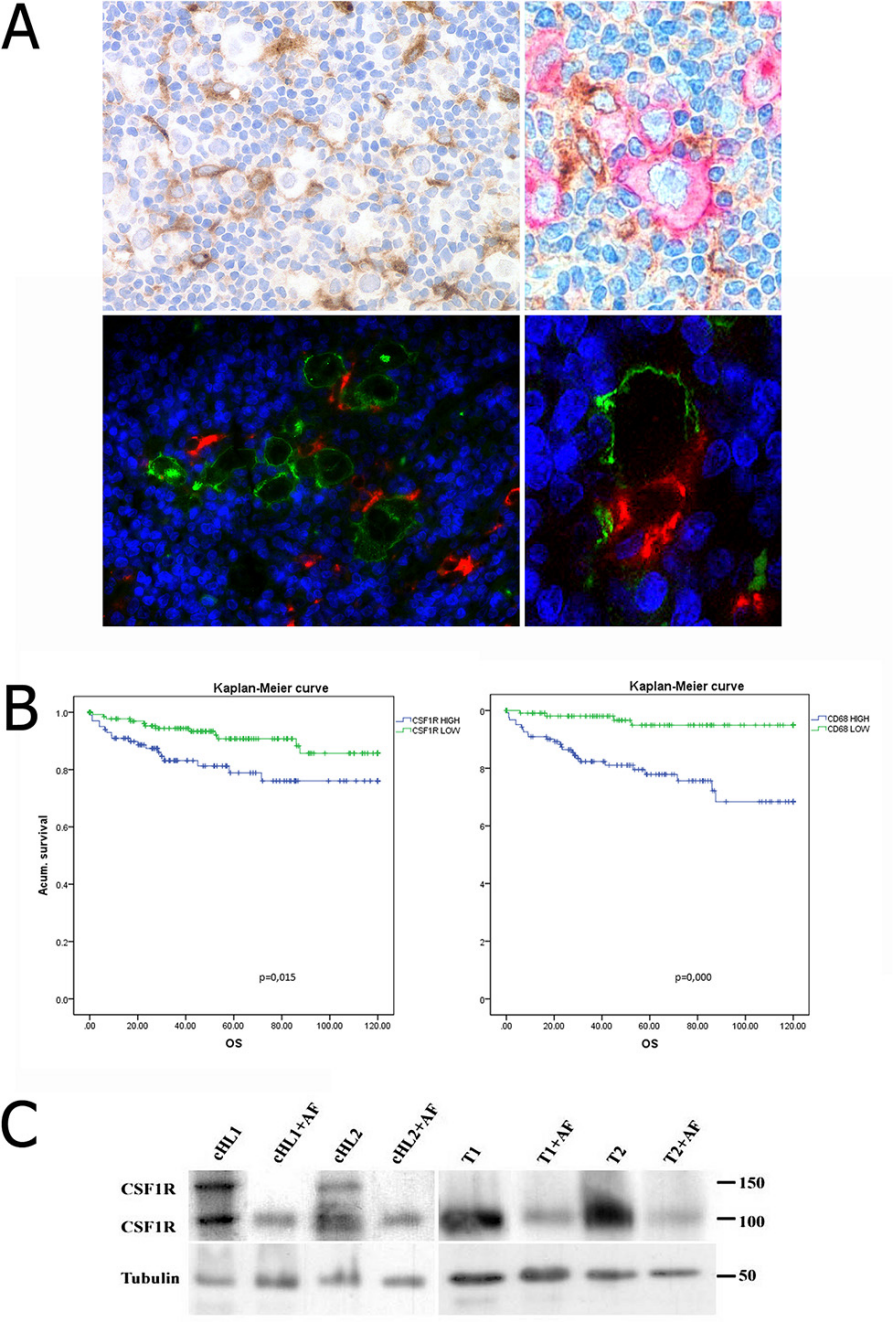
Functional analyses of CSF1R in cHL. (A) Interaction between CSF1R+ macrophages and HRS cells in cHL; above images corresponds to double immunostaining for CSF1R (brown) and CD30 (red), below images corresponds to double immunofluorescence for CSF1R (red) and CD30 (green). While CSFR1 protein expression was observed in cells around HRS, CSFR1 protein was not detected in the CD30+ HRS cells. (B) Kaplan–Meier analyses of survival for CD68 and CSF1R. (C) Western blot analyses of CSF1R protein before and after removal of phosphatase residues with lambda protein phosphatase (AF) using protein extracts from two nodular sclerosis cHL tumour samples and two tonsils. The 150 kDa protein band present in the cHL samples was lost after lambda protein phosphatase treatment.

Detectable CSF1R was either absent or present at negligible levels in the HRS cells (Fig 3). Significant cytoplasmic expression of CSF1R in the HRS cells was only observed in 4 out of 284 cases analysed. This observation is consistent with the results from the WB studies which showed the absence of CSF1R protein in cell lines (Fig 1B). In view of the recent reports describing the expression of CSF1R protein in cell lines [24] and *CSF1R* mRNA in tumour cells of cHL cases [23], double immunoenzymatic and immunofluorescence labelling studies were also performed in cHL tumours (Fig 4A), showing that CD30+ HRS cells lack detectable CSF1R.

To evaluate functional interactions mediated by the receptor, we also compared the levels of CSF1R and CSF1 ligand in cHL cell lines and tumours using Q-PCR. Overall, all cHL cell lines expressed higher levels of *CSF1L* mRNA than *CSF1R* mRNA, in contrast with the whole-tissue sections of the cHL samples, which had higher levels of *CSF1R* mRNA (Figure A in S1 File). This is in agreement with the absence of protein expression observed in all the analysed cell lines (Figure A in S1 File).

The paired t-tests of individual variables were performed to evaluate the relationship between CSF1R+ macrophages and other microenvironment markers. The results are shown in Table C in S1 File. As expected, CSF1R IHC expression was associated with all the other macrophage markers (CD68, CD163, STAT1, and LYZ). Strikingly, CSF1R expression in cHL was also significantly associated with age and EBV status.

Finally, survival analyses showed that CD68 and CSF1R expression were associated with OS (Fig 4B), consistent with previous observations [10,13]. In the multivariate analyses, CD68 expression retained its significant association with OS, but CSF1R did not, using the median of protein expression as a cut-off (Table D in S1 File), further confirming that CSF1R+ cells represent a monocyte/macrophage subpopulation.

### CSF1R pathway activation of cHL

It is of particular note that, in most cases, the CSF1R+ TAMs had a close relationship with the neoplastic HRS cells (Fig 4A), suggesting the existence of some interaction mediated by the receptor between these two cell populations. Of interest was the presence of the two CSF1R proteins of 100 and 150 kDa recognized by antibody FER216 in both WB and double IP experiments in cHL cases (Fig 1B and 1C), that could be related to higher levels of receptor activation in the tumour microenvironment than in that of RLT. To test whether the large sized band in cHL was due to potential phosphorylation, we treated protein extracts from two cHL cases and two tonsils with lambda protein phosphatase prior to western blot. As shown in Fig 4C, this treatment resulted in the removal of the 150 kDa band and supported the phosphorylation of CSF1R.

## Discussion

Despite improvements in treatment strategies more than 20–30% of patients with advanced-stage cHL continue to die from progressive disease. It is, therefore, essential to identify new prognostic markers and also improved therapeutic targets. TAMs have been recently proposed to be a factor that predicts survival in cHL [10,28] and FL [17,20]. An important approach to achieving this is the development and use of properly validated antibody reagents that can be used within a routine clinical context. The current study describes one such reagent recognising CSF1R, which could represent an important tool in the future study of cHL and other lymphomas.

TAMs are one of the many types of tumour-associated monocytic cells that have been described which play a role in tumour development. Others include tumour-infiltrating myeloid cells (TIMs), CD11b (ITGAM) (+) TIMs, and CD11b+ myeloid-derived suppressor cells (MDSC) [29,30](). Not only TAMs have been identified as regulators of solid tumour development based on their capacity to enhance the angiogenic, invasive and metastatic programming of neoplastic tissue [31,32] but the tumour cells themselves can attract peripheral macrophages and re-program these cells to become immunosuppressive cells favouring tumour development [33]. Other factors promoting tumour development include endothelial cells which also provide critical signals for the selective growth and differentiation of macrophages from several hematopoietic progenitors [34]. This endothelial niche for the differentiation and functional polarization of M2-like macrophages creates additional bidirectional links between angiogenesis, macrophage recruitment and polarization. Thus the composition of the tumour microenvironment plays a key role in tumour growth.

CSF1, a ligand of CSF1R, is the main cytokine involved in the recruitment, differentiation, and activation of tissue macrophages, and thus plays an essential role in the recruitment of TAMs [21]. Indeed, CSF1 has been implicated in the control of recruitment and polarization of macrophages in several solid cancers[35,36,37]. CSF1 stimulated cells were initially classified a M2-type cells in contrast to the M1-type macrophages differentiating in response to granulocyte/macrophage CSF with their mainly immunoregulatory roles [36]. However, the finding in this study that the CSF1R+ cell population only partially overlapped with the M2-type macrophages detected by CD163 expression is consistent with previous observations about monocyte differentiation, in which the *CSF1R* gene was not significantly differentially expressed between M1 versus M2 monocyte activation models [27]. The second CSF1R ligand, interleukin 34 (IL-34), also regulates macrophage recruitment to tissues but exhibits a different tissue distribution [38]. However, the exact role of the various ligands/receptor interactions of CSF1R pathway in cancer remains to be clarified.

Those cHL patients possessing a high TAM content, identified using CD68 or CSF1R, showed a trend toward reduced progression free survival and a significantly shorter OS in this study. Although other studies have also reported the value of several macrophage-associated antigens, including CD68, CD163 (marker of M2 macrophages), STAT1, and LYZ, as prognostic markers [10,11,12,23], others have described contradictory results. Such variation may reflect differences in techniques [13] as well as the intrinsic functional heterogeneity of the tissue macrophage populations [39]. Such discrepancies make it difficult to standardize methodologies for evaluating TAMs in clinical practice. A more detailed description of macrophage differentiation and functionalities should help us better understand the pathogenesis of the disease and the biological basis of tumour-macrophage interactions.

No significant CSF1R protein expression was detected in the neoplastic HRS cells of cHLs or in HRS-derived cell lines using IHC, WB, or double IP. This is in accordance with previous reports describing CSF1 (ligand) expression in tumour cells but the receptor, CSF1R, in the surrounding TAM populations [40]. In contrast, a recent report describes some level of CSF1R protein expression in a fraction of HRS cells in cHL [41], but more generalized CSF1R protein expression was associated with the microenvironment, using a polyclonal antibody. Also, the presence of *CSF1R* transcripts have been described in the HRS cells [23,24]. Possible explanations for the differences between our results and those in previous studies include the use of reagents with different specificities and other methodologies as described above.

The results showing the presence of a hyperphosphorylated CSF1R protein in cHL cases highlight the importance of CSF1/CSF1R signalling in the recruitment of TAMs, and the importance of this pathway in the reciprocal crosstalk between tumour HRS cells and the microenvironment. Previous studies have detected anti-tumour activity after disrupting the CSF1/CSF1R signalling pathway, probably as a result of blocking the recruitment and inhibiting the activation of TAMs [42,43]. Indeed, small molecule anti-CSF1R drugs are now being tested in several tumour models [44,45]. Moreover, use of an anti-CSF1R antibody depletes TAMs in animal models and also in human tumours [46], also suggesting therapeutic potential in cancer. Results from the current paper showing evidence of close physical interactions between CSF1R+ TAMs and tumour HRS cells and phosphorylation, and therefore activation, of the CSF1R pathway in cHL tumour tissues provides further evidence of an important role for this pathway in cHL.

In conclusion, the use of a new completely validated mAb able to be used in routine laboratory procedures has enabled the study of CSF1R protein expression in normal lymphoid tissue, a variety of NHLs, and cHL. The expression of CD68 and CSF1R on TAMs was associated with decreased OS in cHL and CSF1R was found to be hyperphosphorylated in cHL. The availability of IHC techniques for detecting CSF1R protein and other members of the CSF1R/CSF pathway in tissues represent potential valuable approaches to identify future biomarkers and also therapeutic targets for high risk cHL.

## Supporting Information

S1 File Contains supplementary materials and results, organized in Tables A-D and Figure A, and supplementary references. Table A in S1 File. Clinical and pathological data of the cHL patients included in the survival analyses. Table B in S1 File. Antibodies used in the study. Table C in S1 File. Relationships between clinical characteristics and immunohistochemical variables. IHC protein expression was quantified using an automated scan, Chroma Vision Systems- ACIS III (DAKO, Glostrup, Denmark), analysing the whole area of representative tissue included in the TMAs. Associations were analysed using the Spearman test (continuous variables) or the Pearson chi-square test (categorical variables). P values are shown, grey boxes indicating significant associations (p<0.05). EXTRANODAL: extranodal disease. IPS: International Prognostic Score. STAGE IV, stage IV disease (according to the Ann Arbor classification). HB: haemoglobin level <10.5 g/dl. ALB: Serum albumin level <4 g/dl. LEUK, leucocytosis (leukocyte count >15,000/mm3). LYMPH, lymphocytopenia (lymphocyte count <600/mm3, or 8% of the leukocyte count, or both). AGE: age 45 years or older. SEX: male. DOD, death from disease. OS, overall survival (months). FFS, failure-free survival (months). OUTCOME, unfavourable versus favourable treatment response (treatment failure of first line therapy versus complete remission without relapse). Table D in S1 File. Multivariate Cox analyses. Figure A in S1 File. Western blot and Q-PCR analyses of CSF1R. (A) Western blot analyses of CSF1R protein using protein extracts from HEK293 cells transfected with either CSF1R or a closely related protein (FGFR1) as a negative control, normal tissues, cHL tumour samples, and cHL-derived cell lines. Antibody FER216 recognised a band of 100 kDa in tonsil extracts and two bands of 100 and 150 kDa in extracts from three cHL biopsies. The mAb to tubulin was used as the loading control. (B) Q-PCR detection of CSF1R and CSF1R ligand (CSF1L) mRNA from cHL tumour samples, and cHL-derived cell lines (the y-axis represents the normalized relative levels).
(DOC)
