# Supplementary material for: CSF1R Protein Expression in Reactive Lymphoid Tissues and Lymphoma: Its Relevance in Classical Hodgkin Lymphoma
Source: PLoS One. 2015 Jun 12;10(6):e0125203. doi: 10.1371/journal.pone.0125203 (PMC4466308; doi:10.1371/journal.pone.0125203)
Supplement: S1 File — Table A in S1 File. Clinical and pathological data of the cHL patients included in the survival analyses. Table B in S1 File. Antibodies used in the study. Table C in S1 File. Relationships between clinical characteristics and immunohistochemical variables. IHC protein expression was quantified using an automated scan, Chroma Vision Systems- ACIS III (DAKO, Glostrup, Denmark), analysing the whole area of representative tissue included in the TMAs. Associations were analysed using the Spearman test (continuous variables) or the Pearson chi-square test (categorical variables). P values are shown, grey boxes indicating significant associations (p<0.05). EXTRANODAL: extranodal disease. IPS: International Prognostic Score. STAGE IV, stage IV disease (according to the Ann Arbor classification). HB: haemoglobin level <10.5 g/dl. ALB: Serum albumin level <4 g/dl. LEUK, leucocytosis (leukocyte count >15,000/mm3). LYMPH, lymphocytopenia (lymphocyte count <600/mm3, or 8% of the leukocyte count, or both). AGE: age 45 years or older. SEX: male. DOD, death from disease. OS, overall survival (months). FFS, failure-free survival (months). OUTCOME, unfavourable versus favourable treatment response (treatment failure of first line therapy versus complete remission without relapse). Table D in S1 File. Multivariate Cox analyses. Figure A in S1 File. Western blot and Q-PCR analyses of CSF1R. (A) Western blot analyses of CSF1R protein using protein extracts from HEK293 cells transfected with either CSF1R or a closely related protein (FGFR1) as a negative control, normal tissues, cHL tumour samples, and cHL-derived cell lines. Antibody FER216 recognised a band of 100 kDa in tonsil extracts and two bands of 100 and 150 kDa in extracts from three cHL biopsies. The mAb to tubulin was used as the loading control. (B) Q-PCR detection of CSF1R and CSF1R ligand (CSF1L) mRNA from cHL tumour samples, and cHL-derived cell lines (the y-axis represents the normalized relative levels). (DOC) [file pone.0125203.s001.doc]

**CSF1R protein expression in reactive lymphoid tissues and lymphoma: Its relevance in classical Hodgkin lymphoma**

Ana M. Martín-Moreno^1^*, Giovanna Roncador^2^*, Lorena Maestre^2^, Elena Mata^1^, Scherezade Jiménez^2^, Jorge L. Martínez-Torrecuadrada^3^, Ana I. Reyes-García^2^, Carmen Rubio^1^, José F. Tomás^4^, Mónica Estévez^4^, Karen Pulford^5^, Miguel A. Piris^6^ and Juan F. García^1^.

# Supplementary information

## Protein and monoclonal antibody production

An episomal expression system based on Epstein-Barr virus (EBV) components was used for recombinant CSF1R production in mammalian cells (HEK293). To this end, the sequence encoding the extracellular domain of CSF1R (NP_005202.2, residues 1-514) was fused to a C-terminal human IgG1 Fc sequence in a CMV-based pTT3 vector which features the EBV oriP replicator to allow episomal replication of the plasmid within cells [45]. The protein was produced transiently in HEK293-EBNA cells, expressing the EBV nuclear antigen (EBNA)1 that drives episomal replication of oriP containing plasmids, by transfection using FuGENE6 Transfection Reagent (Promega, Madison, Corporation). After 5 days post-transfection, secreted CSF1R-Fc was purified from the clarified growth media using a Protein A column (GE Healthcare) connected to an ÄKTA-prime system (GE Healthcare). The purified fusion protein was buffer-exchanged into PBS by gel filtration using a HiPrep 26/10 Desalting column (GE Healthcare) on an ÄKTAFPLC system (GE Healthcare) and concentrated by Vivaspin ultrafiltration devices with a molecular weight cutoff of 10 kDa (Vivascience, USA).

Two BALB/c mice were injected intraperitoneally (three times at 14-day intervals) with 100 μg of CSF1R-Fc fusion protein and Complete Freund's adjuvant (Difco). A 150-μg booster of the recombinant CSF1R-Fc protein was injected intraperitoneally, and fusion was performed 3 days later, as described [46].

Hybridoma supernatants were screened by ELISA and using HEK-293T cells transfected with pCDNA3-Fc-CSF1R plasmid. The mouse mAb that was raised against CSF1R (clone FER216, IgG1) was cloned by the limiting dilution technique. Animal experiments were performed under the experimental protocol approved by the Institutional Committee for Care and Use of Animals.

To confirm that clone FER216 recognized the human CSF1R protein, immunohistochemistry was performed on frozen cytospin preparations of Fc-tagged human CSF1R expressed in HEK293 (Figure A, panel A). Cytospin preparations of Fc-tagged human FGFR1 protein (Fibroblast Growth Factor Receptor 1, a closely related receptor of the same Fms-Like Tyrosine Kinase family) were used as a negative control. Labeling with the anti-Fc mAb confirmed the efficiency of transfection.

## Table A. Clinical and pathological data of the cHL patients included in the survival analyses.

| **Variable** | **Nº cases** | **%** |
| --- | --- | --- |
| **Age** |  |  |
| > 45 | 75 | 30.1 |
| < 45 | 174 | 69.9 |
| **Stage** |  |  |
| II-III | 184 | 73.9 |
| IV | 65 | 26.1 |
| **IPS** |  |  |
| ≥ 3 | 162 | 65 |
| < 3 | 87 | 35 |
| **CSF1R *** |  |  |
| High | 105 | 42.1 |
| Low | 144 | 57.9 |
| **cHL subtype** |  |  |
| Nodular sclerosis cHL | 162 | 65 |
| Mixed cellularity cHL | 68 | 27.4 |
| Lymphocyte-rich cHL | 19 | 7.6 |
| **Total** | **249** |  |

* High vs. low means higher vs. lower than the mean expression across the whole series (value=27.5)

## Table B. Antibodies used in the study

| **Molecule** | **Antibody type** | **Clone name** | **Dilution** | **Source** |
| --- | --- | --- | --- | --- |
| CSF1R | Mouse monoclonal | FER216 | 1:10 supernatant | CNIO |
| CSF1R | Mouse monoclonal | 61701 | 1:50 | R&D System |
| CD30 | Mouse monoclonal | CON6D | 1:10 | CNIO |
| CD68 | Mouse monoclonal | PGM1 | 1:1000 | Dako |
| CD163 | Mouse monoclonal | 10D6 | 1:40 | Novocastra |
| Fc | Rat monoclonal | ARI358D | 1:20 supernatant | CNIO |
| FOXP3 | Mouse monoclonal | 236A | 1:4 supernatant | CNIO |
| PD-1 | Mouse monoclonal | NAT105 | 1:4 supernatant | CNIO |
| STAT1 | Mouse monoclonal | STAT1 | 1:100 | Santa Cruz Biotechnology |
| LYZ | Rabbit polyclonal | EC 3.2.1.17 | 1:300 | Dako |
| Granzyme B | Mouse monoclonal | GrB-7 | 1:10 | Dako |

## Double immunenzymatic staining

For paraffin embedded tissues an initial automated dewaxing and rehydration step followed by heat-induced (100°C for 20 min) or enzyme-induced antigen retrieval (10 to 15 min, Bond Enzyme Pretreatment Kit, Leica Biosystems) was performed. Heat-induced antigen retrieval was performed using pH 8.8 ethylenediaminetetraacetic acid (EDTA)-based ready-to-use solution (Leica Biosystems). The slides were subsequently incubated with 3% hydrogen peroxide (5 min), primary CSF1R (FER216) antibody (30 min), a post-primary blocking reagent (to prevent nonspecific polymer binding) (8 min), horseradish peroxidase (HRP)-labeled rabbit anti-mouse Ig polymer (8 min), and diaminobenzidine substrate (10 min). All reagents were components of the Bond Polymer Refine detection system (Leica Biosystems). A second immuno-alkaline phosphatase (AP) procedure was then performed, omitting the dewaxing, rehydration, and epitope retrieval steps. The primary antibodies CD68, CD163, and CD30 were applied for 40 min followed by incubation with post-primary AP blocking reagent (20 min) and AP-labeled rabbit anti-mouse Ig (30 min), both of which are components of the Bond Polymer AP Red detection system (Leica Biosystems). The AP reaction was developed with either the Fast Red substrate included in the Bond Polymer AP Red detection system. Hematoxylin counterstaining was performed.

## Immunoperoxidase technique combined with immunofluorescence

After performing the initial CSF1R IHC staining procedure with HRP rabbit anti-mouse Ig polymer (8 min), and diaminobenzidine substrate (10 min) as described above, the sections were then incubated for 45 minutes at room temperature with CD68, CD163 or CD30. Sections were washed in PBS for up to 5 minutes and then incubated in the dark for 45 minutes with secondary antibodies (specific for species, isotypes or subclass) and labelled with contrasting green and red fluorochromes (Alexa Fluor 488 and Alexa Fluor 594, dilution 1:200). The slides were washed in PBS for up to 5 minutes. Following washing, antifading (Qbiogene, Illkirch, FR) and DAPI (Molecular Probes, Leiden Netherlands) were added.

Slides were examined on a Nikon E800 Eclipse fluorescence microscope (Nikon, Kingston-upon-Thames, UK) equipped for epifluorescence. Fluorescence images were captured with an Axiocam charge-coupled device (CCD) camera (Zeiss, Jena, Germany) and Axiovision software (Imaging Associates, Bicester, UK), and adjusted using Photoshop software (Adobe, San Jose, CA, USA). The immunoperoxidase image was viewed by transmitting light, and corresponding grey-scale images were inverted and pasted into the red (for CSF1R) channel of the red-green-blue (RGB) Photoshop image. By means of this technique, cells expressing CSF1R were visualized in red, whereas immunofluorescence labeling marker was detected in green.

## Non isotopic in-situ hybridization

Non-isotopic in situ hybridization for the Epstein–Barr virus (EBV) early RNA (EBER) was carried out using a fluorescein-conjugated PNA probe (DAKO) and an anti-FITC antibody (clone DAK-FITC4, DAKO), following the manufacturer’s recommendations.

## Western blot (total protein extraction and blot visualization)

Western blot was performed with cell extract of human tonsil, testicle, and 3 cases of nodular sclerosis cHL and 5 cHL human cell lines (L428, L1236, KM-H2, HDLM2 and L540) (Figure A, panel B). To extract total protein, cells were lysed in a buffer containing 50 mM Tris (tris (hydroxymethyl) aminomethane)-HCl, pH 7.5, 150 mM NaCl, 1% Igepal (Sigma Chemical) and protease inhibitors (Roche, Mannheim, Germany). Lysates were incubated in a cool room on a rotary shaker for 1 h and cleared by centrifugation. The total lysates of each cell line were denatured by heating in Laemmli sample buffer, resolved on a 10% sodium dodecyl sulfate-polyacrylamide gel (SDS-PAGE) and transferred onto nitrocellulose membranes for 2 h. Membranes were incubated overnight with blocking solution (5% milk in PBS) and immunoblotted for 1 h at room temperature with anti-CSF1R (FER216) (diluted 1:10),anti-CSF1R (61701) and α-tubulin monoclonal antibody (1:500) (CNIO), followed by incubation with horseradish peroxidase-conjugated secondary antibody (DAKO, Glostrup, Denmark). Finally, the blots were visualized using the ECL detection system (Amersham Biosciences, Buckinghamshire, UK) in accordance with the supplier’s instructions.

A specific band of 107 kDa was observed in HEK-CSF1R-Fc, tonsil and the in the 3 cHL cases (Figure A, panel B). CSF1R protein was not detected in HEK-FGFR1-Fc, testicle or in the 5 cHL cell lines (L428, L1236, KM-H2, HDLM2 and L540).

## Immunoprecipitation

The specificity of clone FER216 for human CSF1R protein was confirmed by using this antibody in western blotting of material that had been previously immunoprecipitated from tonsil, 1 nodular sclerosis cHL case and 2 cHL cell lines (KM-H2 and L540) lysates using either FER216 mAb or the CSF1R monoclonal antibody (Clone 61701) from R&D Systems. 300ug of lysate soluble proteins were incubated with 40 µl of either FER216 mAb supernatant or 2 µg of Clone 61701 mAb overnight at 4 C. Tagged proteins were immunoprecipitated with protein G-Sepharose (Santa Cruz Biotechnology, CA, USA), and immunocomplexes were washed four times with HNTG buffer (20 mM Hepes pH 7.5, 150 mM NaCl, 0.1% Triton x-100, 10% glycerol). As shown in Table A

igure A, panel C, both antibodies immunoprecipitated a protein with a molecular weight of 107 kDa in tonsil and in the cHL and a second band of around 150 kDa in cHL which was recognized in western blotting by the other antibody thus confirming recognition of CSF1R by both FER216 and Clone 61701.

## Quantitative reverse transcriptase polymerase chain reaction (Q-PCR)

For Q-PCR, RNA was extracted from 5 cHL tumor samples and tonsils (frozen tissues), and cHL cell lines, using an RNAeasy mini kit (Qiagen, Venlo, Limburg, Netherlands). After DNAse digestion, first-strand complementary DNA was synthesized from total RNA using random primers and a High Capacity cDNA Reverse Transcription Kit (Life Technologies, Carlsbad, CA, USA). Gene expression of CSF1R and CSF1 ligand were analyzed by using TagMan gene expression assays (CSF1L Hs00174164_m1; CSF1R Hs00911250_m1) and GUSB as endogenous control. All reactions were performed using a CFX96 thermal cycler (Bio-Rad, Hercules, CA, USA), and relative gene expression levels were assessed by the delta cycle threshold (CT) method.

**Figure A. Western blot and Q-PCR analyses of CSF1R.**

(A) Western blot analyses of CSF1R protein using protein extracts from HEK293 cells transfected with either CSF1R or a closely related protein (FGFR1) as a negative control, normal tissues, cHL tumor samples, and cHL-derived cell lines. Antibody FER216 recognised a band of 100 kDa in tonsil extracts and two bands of 100 and 150 kDa in extracts from three cHL biopsies. The mAb to tubulin was used as the loading control. (B) Q-PCR detection of CSF1R and CSF1R ligand (CSF1L) mRNA from cHL tumor samples, and cHL-derived cell lines (the y-axis represents the normalized relative levels).


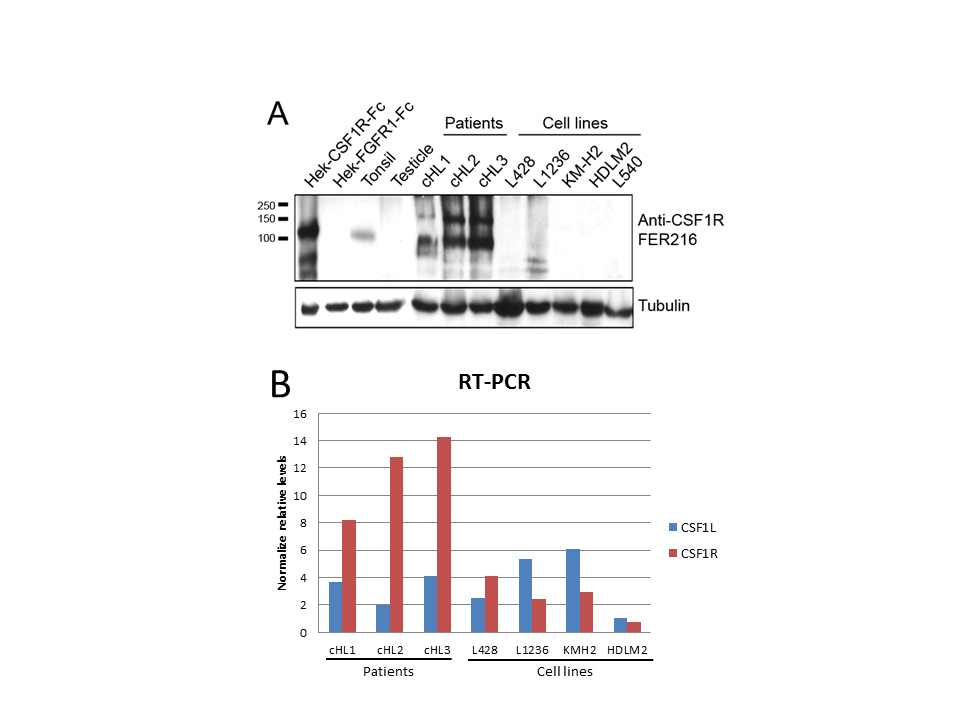


**In-gel digestion and LC-MS/MS analysis**

We performed In-gel digestion and LC-MS/MS analysis to identify the 100k-Da protein band detected by IP in tonsil and in the cHL3 case, and the additional 150 kDa band present in the cHL3 case.

Bands corresponding to 100 and 150 kDa, were excised from the gels, washed, reduced and alkylated before digestion with proteomic grade trypsin (Promega, Madison, WI, USA) dried using a speed-vac and re-suspended in 0.1% formic acid in LC MS-grade water (Sigma Aldrich, St Louis, MI, USA) [47]. Eluted peptides were separated by reversed-phase chromatography (Reprosil-Pur C18 3 µm, 200*0.075 mm, Dr. Maisch GmbH), using a nanoLCUltra1D+ system (Eksigent, Dublin CA, USA), directly coupled online with a LTQ-OrbitrapVelos instrument (Thermo Fisher Scientific, Waltham MA, USA).

Mass spectra were acquired in a data-dependent manner, with an automatic switch between MS and MS/MS scans using a top 15 method with a threshold signal of 1000 counts. MS spectra were acquired with a resolution of 60000 (FWHM) at 400 m/z in the Orbitrap. Peptide fragmentation was performed with collision-induced dissociation (CID). To avoid repeatedly sequencing the most abundant peaks eluting from the column we established a dynamic exclusion window of 70s.

The fragmentation spectra were searched against the Uniprot_Mouse database, comprising both the canonical and manually reviewed isoform sequences (release 2012_09, 50544 entries) using MASCOT (v 2.2) 3 as the search engine [48]. Only peptides with an FDR <1 % were considered for protein identification. The protein detected in the 107 and 150 kDa bands were identified as CSF1R.

**Phosphate removal with lambda protein phosphatase (lambda PP)**

A lambda protein phosphatase protocol able to remove the phosphatase residues was used to determine the phosphorylation status of the receptor. Protein extracts were prepared from two frozen samples of nodular sclerosis cHL and two tonsils as normal reactive control. Forty µg of each protein extract were treated with lambda PP (Upstate-Millipore, Darmstadt, Germany) in 5 µL of lambda protein phosphatase buffer containing 5 mM DTT (DL-Dithiothreitol) and lambda protein phosphatase, following the manufacturer’s instructions. After 24 h incubation at 37º C, the reaction was stopped by adding the same volume of SDS sample buffer and the samples were analyzed on a 7.5% SDS-PAGE [49].

**Table C. Relationships between clinical characteristics and immunohistochemical variables.**

IHC protein expression was quantified using an automated scan, Chroma Vision Systems- ACIS III (DAKO, Glostrup, Denmark), analysing the whole area of representative tissue included in the TMAs. Associations were analysed using the Spearman test (continuous variables) or the Pearson chi-square test (categorical variables). P values are shown, grey boxes indicating significant associations (p<0.05). EXTRANODAL: extranodal disease. IPS: International Prognostic Score. STAGE IV, stage IV disease (according to the Ann Arbor classification). HB: haemoglobin level <10.5 g/dl. ALB: Serum albumin level <4 g/dl. LEUK, leukocytosis (leukocyte count >15,000/mm3). LYMPH, lymphocytopenia (lymphocyte count <600/mm3, or 8% of the leukocyte count, or both). AGE: age 45 years or older. SEX: male. DOD, death from disease. OS, overall survival (months). FFS, failure-free survival (months). OUTCOME, unfavorable versus favorable treatment response (treatment failure of first line therapy versus complete remission without relapse).

**Table D**. Multivariate Cox analyses

|  | **B** | **SE** | **Wald statistic** | **df** | **p** | **Exp(B)** | **95% CI of Exp(B)** | |
| --- | --- | --- | --- | --- | --- | --- | --- | --- |
|  |  |  |  |  |  |  | **Low** | **High** |
| **CD68** | 1.270 | 0.396 | 10.294 | 1 | 0.001 | 3.561 | 1.639 | 7.736 |
| **CSF1R** | 0.591 | 0.381 | 2.405 | 1 | 0.121 | 1.805 | 0.856 | 3.807 |
